# Supplementary material for: TMEM106B drives lung cancer metastasis by inducing TFEB-dependent lysosome synthesis and secretion of cathepsins
Source: Nat Commun. 2018 Jul 16;9:2731. doi: 10.1038/s41467-018-05013-x (PMC6048095; doi:10.1038/s41467-018-05013-x)
Supplement: Supplementary file 1 — Supplementary Information [file 41467_2018_5013_MOESM1_ESM.pdf]

Supplementary Information.

TMEM106B drives lung cancer metastasis by inducing TFEB-dependent lysosome synthesis and secretion of cathepsins.

Kundu et al.

Supplementary Figure 1

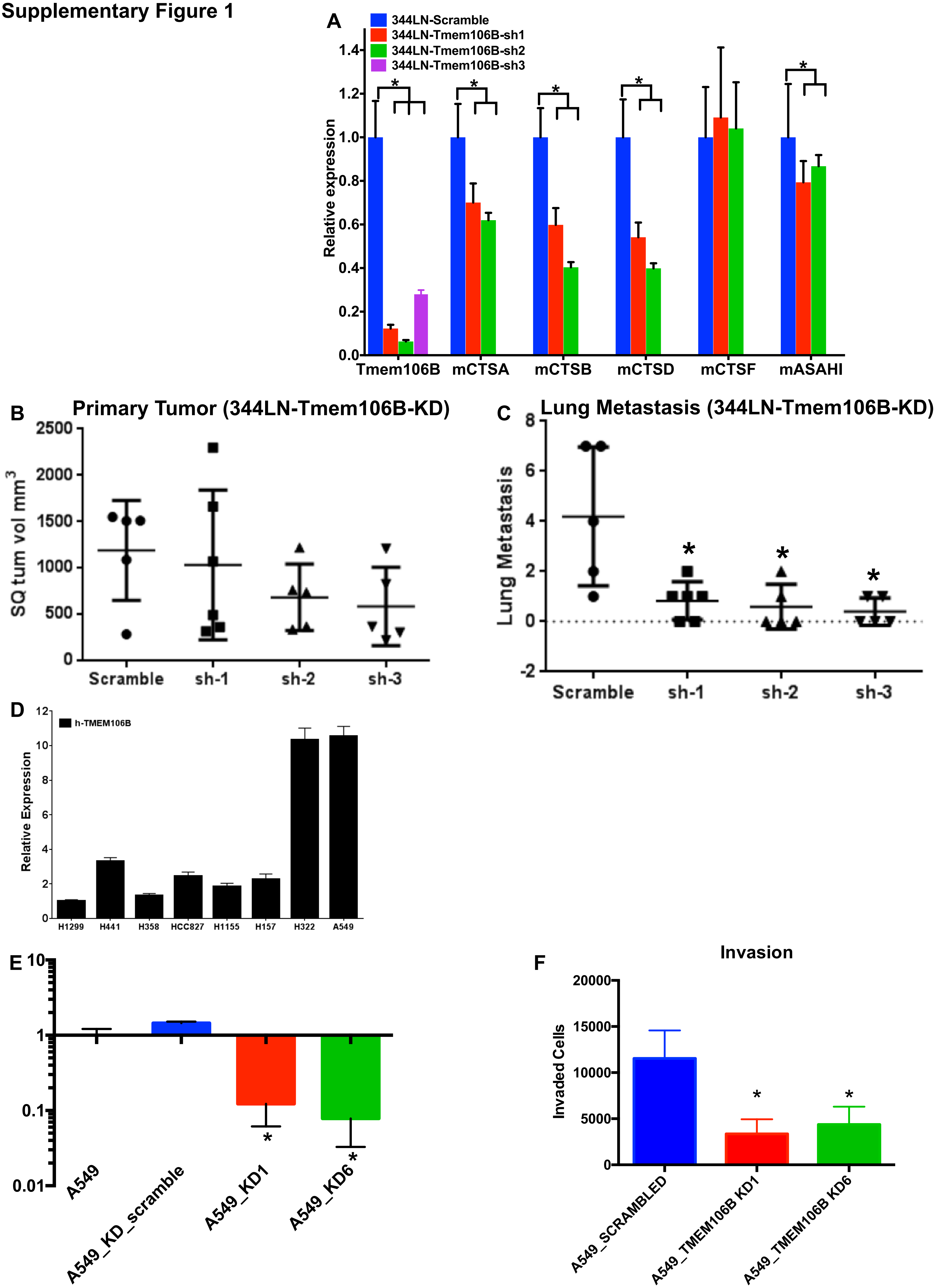

Supplementary Figure 1.

TMEM106B knockdown in mouse and human cells.

(A) qPCR analysis for expression of lysosomal genes upon knock down of TMEM106B expression in mouse 344LN cells. (B) The knock down cells were injected subcutaneously into syngeneic hosts and observed for primary tumor growth and (C) formation of metastatic nodules in lungs. (D) qPCR analysis for assessing expression of endogenous TMEM106B in different human NSCLC cells lines. (E) qPCR analysis for expression of TMEM106B upon knock down of TMEM106B in human A549 cells. (F) The TMEM106B knockdown A549 cells demonstrate reduced ability to invade compared to Scramble controls. All asterisks indicate statistical significance All asterisks indicate statistical significance by T test ($n \geq 3$, * = $p \leq 0.05$).

Supplementary Figure 2

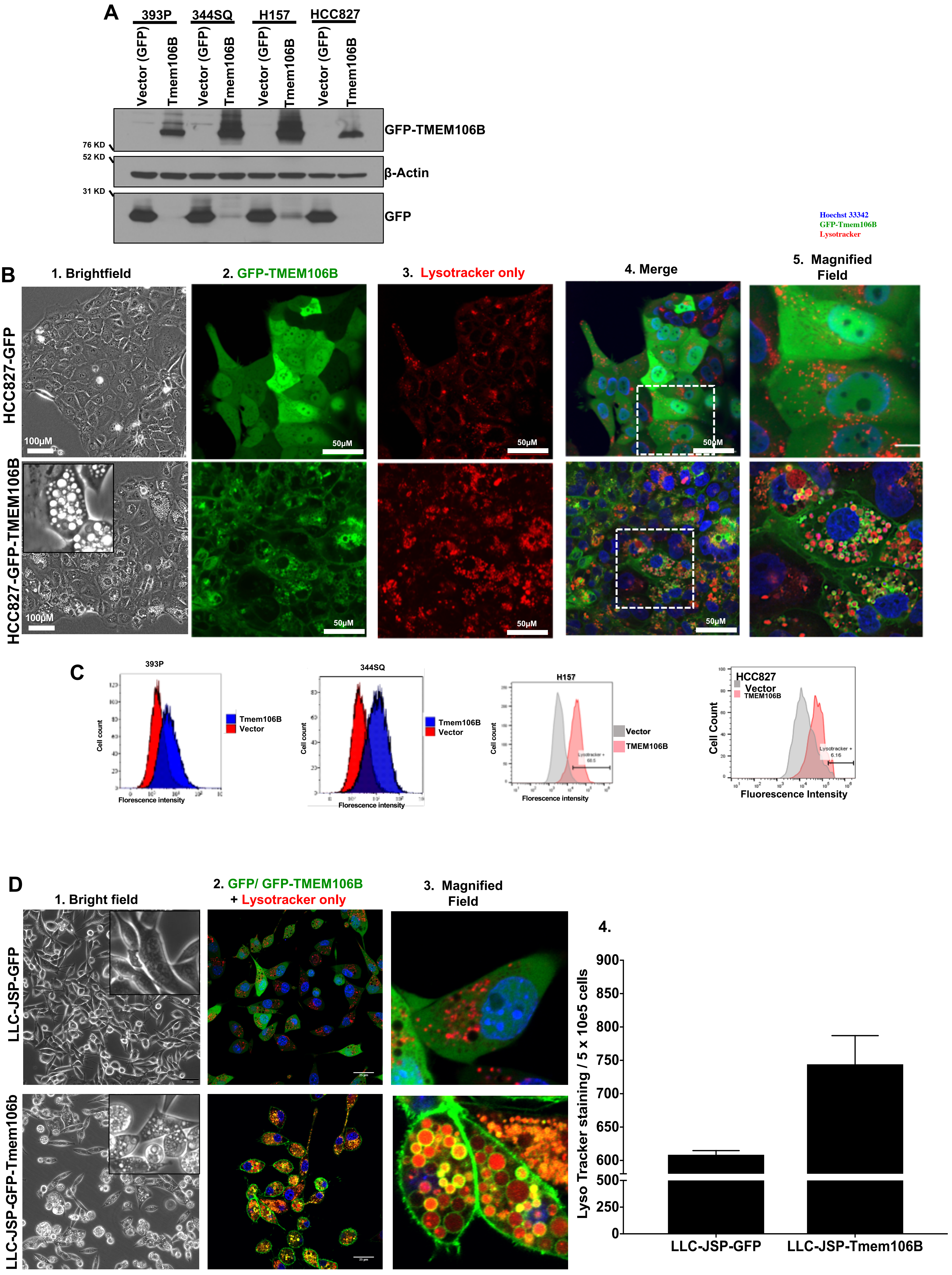

Supplementary Figure 2.

TMEM106B induces synthesis of increased number and size of acidified lysosomes.

(A) Western blot analysis upon 24-48 hr induction of GFP-TMEM106B or GFP alone as control, in the different mouse or human cells. Actin serves as loading control. (B) Human HCC827 lung cancer cells induced to express either GFP only as control, or TMEM106B were imaged as indicated. (1) Cells imaged in bright field to show enlarged vesicular structures formed upon induction of TMEM106B. (2-5) Cells expressing GFP only or GFP tagged TMEM106B were incubated with LysoTracker stain and counter stained with Hoechst33342 were imaged live in real time as indicated. TMEM106B expressing cells form enlarged lysosomes that stain positive for LysoTracker. Magnified fields exhibit the stained vesicles. (C) Lysotracker Stained cells were trypsinized and analyzed by flow cytometer and mean florescence intensity for LysoTracker stained cells were recorded. (D) Mouse LLC-JSP lung cancer cells induced to express either GFP only as control, or TMEM106B were imaged as indicated. (1) Cells imaged in bright field to show enlarged vesicular structures formed upon induction of TMEM106B. (2-3) Cells expressing GFP only or GFP tagged TMEM106B were incubated with LysoTracker stain and counter stained with Hoechst33342 were imaged live in real time as indicated. TMEM106B expressing cells form enlarged lysosomes that stain positive for LysoTracker. Magnified fields exhibit the stained vesicles. (4) Stained cells were trypsinized and analyzed by measuring mean florescence intensity for LysoTracker using a fluorimeter. All asterisks indicate statistical significance All asterisks indicate statistical significance by T test ($n \geq 3$, * = $p \leq 0.05$).

Supplementary Figure 3

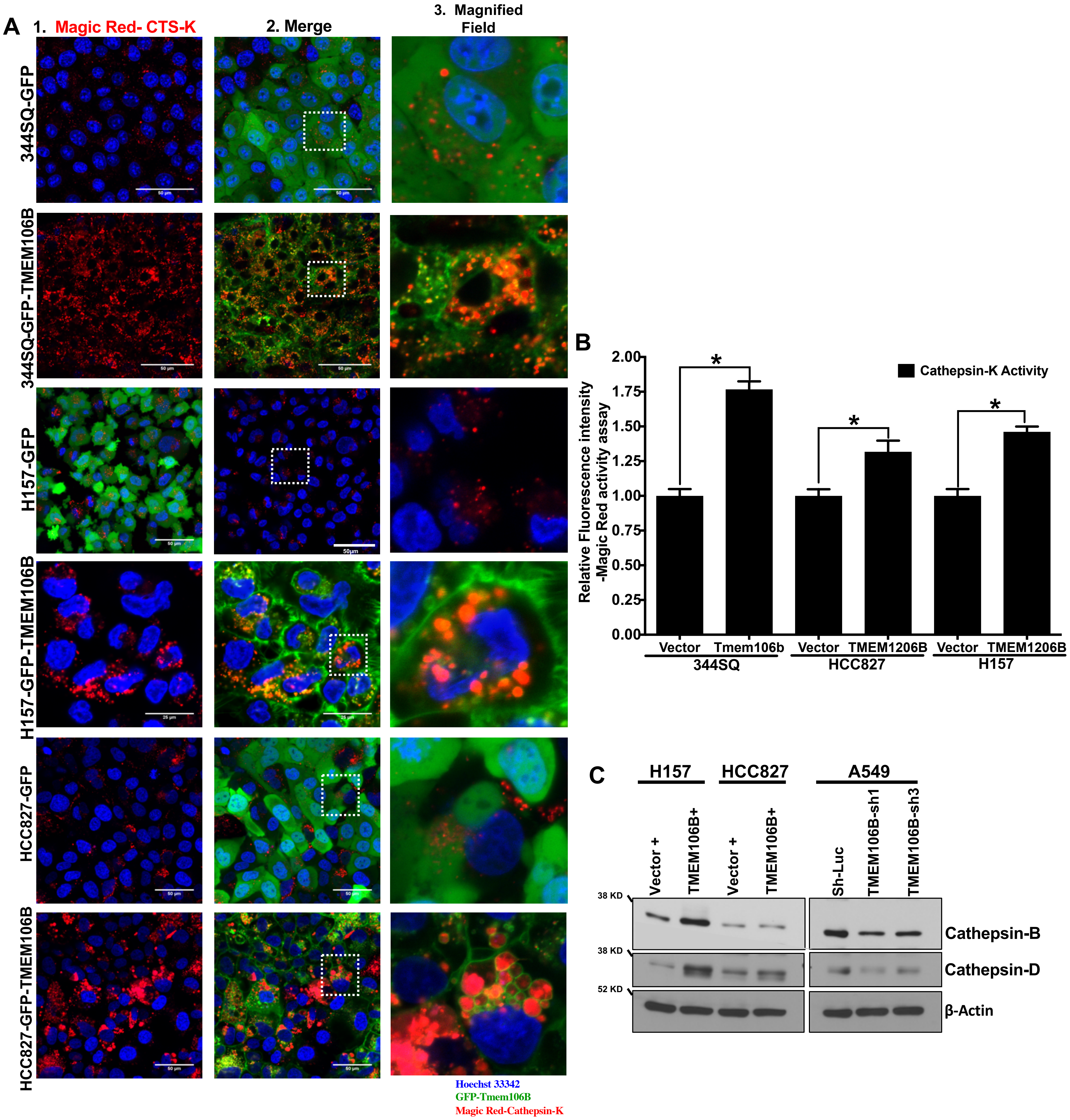

Supplementary Figure 3 contd.

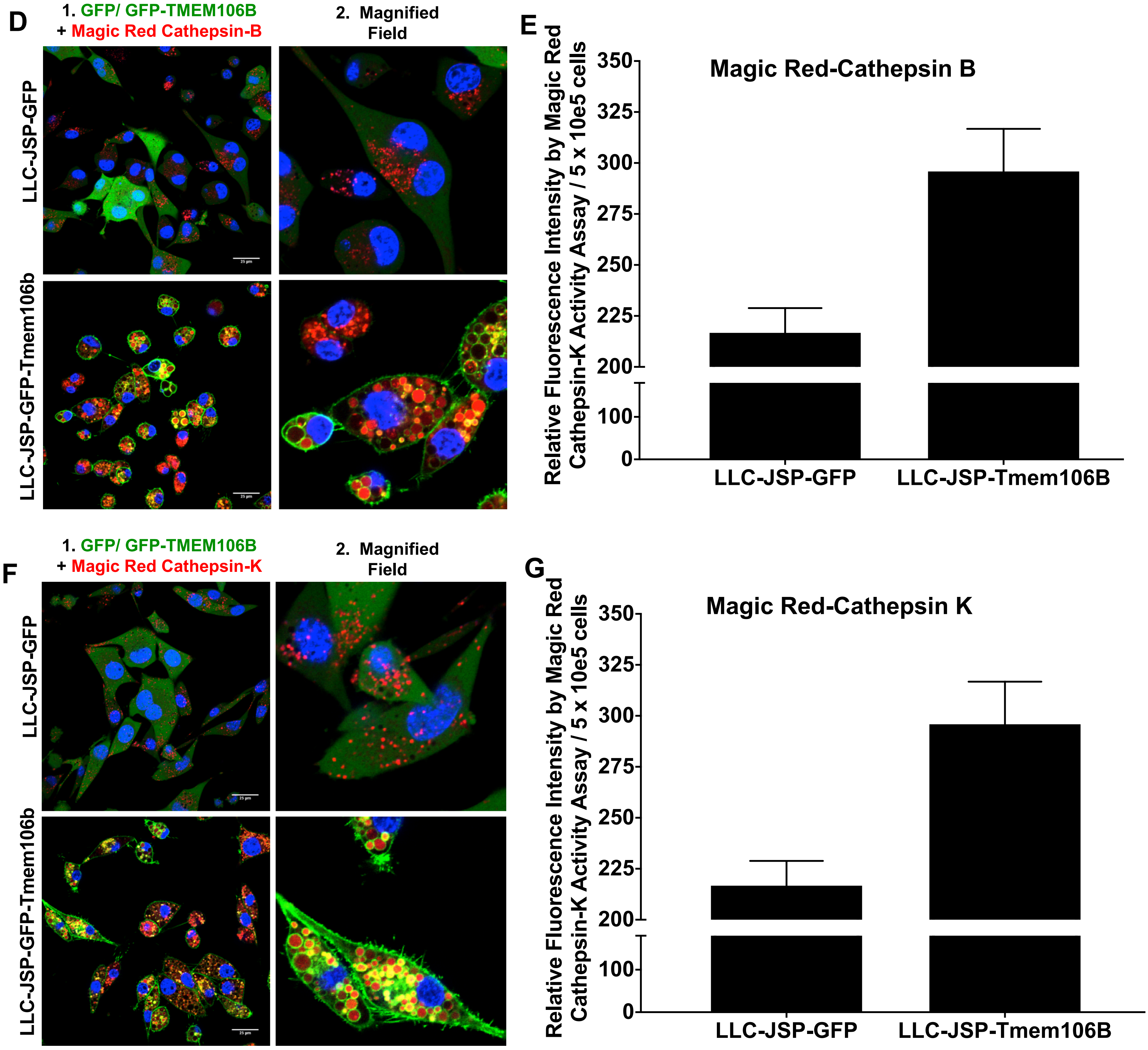

Supplementary Figure 3.

TMEM106B induces lysosomes, that are loaded with catalytically functional cathepsins.

(A) Cells expressing GFP only or GFP tagged TMEM106B were incubated with Magic Red-Cathepsin-K reagent to stain for activity of cathepsin-K and counter stained with Hoechst33342. Cell were imaged live in real time as indicated. (B) Quantification of fluorescence intensity corresponding to cathepsin-K activity as analyzed by Magic Red-Cathepsin-K activity assay in different cells as indicated. (C) Western blot analysis for expression of active cathepsins in human cells with induced expression or knockdown of TMEM106B. (D) LLC-JSP Cells expressing GFP only or GFP tagged TMEM106B were incubated with Magic Red-Cathepsin-B reagent to stain for activity of cathepsin-B and counter stained with Hoechst33342. Cell were imaged live in real time as indicated. (E) Quantification of fluorescence intensity corresponding to cathepsin-B activity as analyzed by Magic Red-Cathepsin-B activity assay in different cells as indicated. (F) LLC-JSP Cells expressing GFP only or GFP tagged TMEM106B were incubated with Magic Red-Cathepsin-K reagent to stain for activity of cathepsin-K and counter stained with Hoechst33342. Cell were imaged live in real time as indicated. (G) Quantification of fluorescence intensity corresponding to cathepsin-K activity as analyzed by Magic Red-Cathepsin-K activity assay in different cells as indicated. All asterisks indicate statistical significance All asterisks indicate statistical significance by T test ($n \geq 3$, * = $p \leq 0.05$).

Supplementary Figure 4

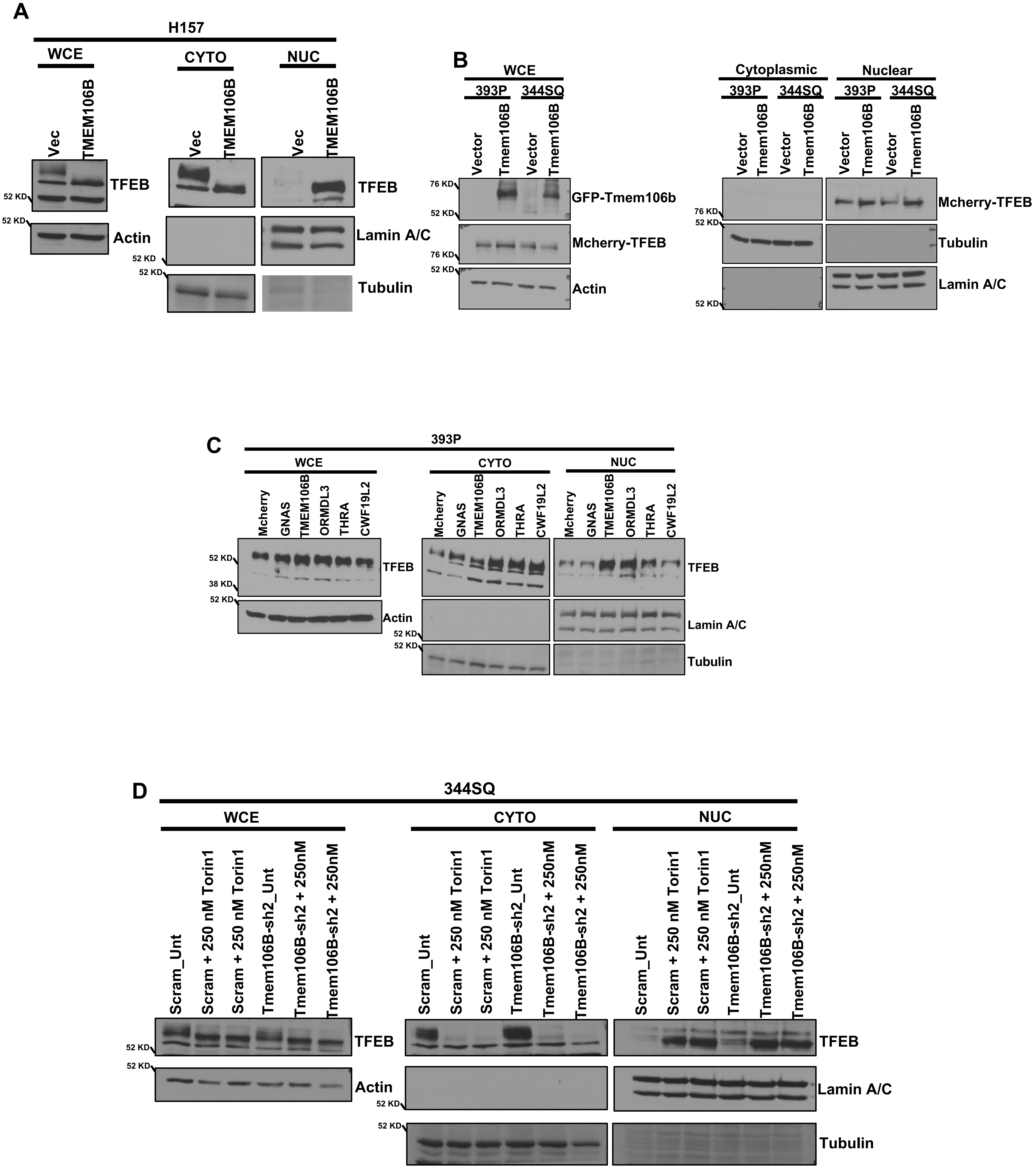

Supplementary Figure 4 cont.

Lysosomal genes on TMEM106B OE

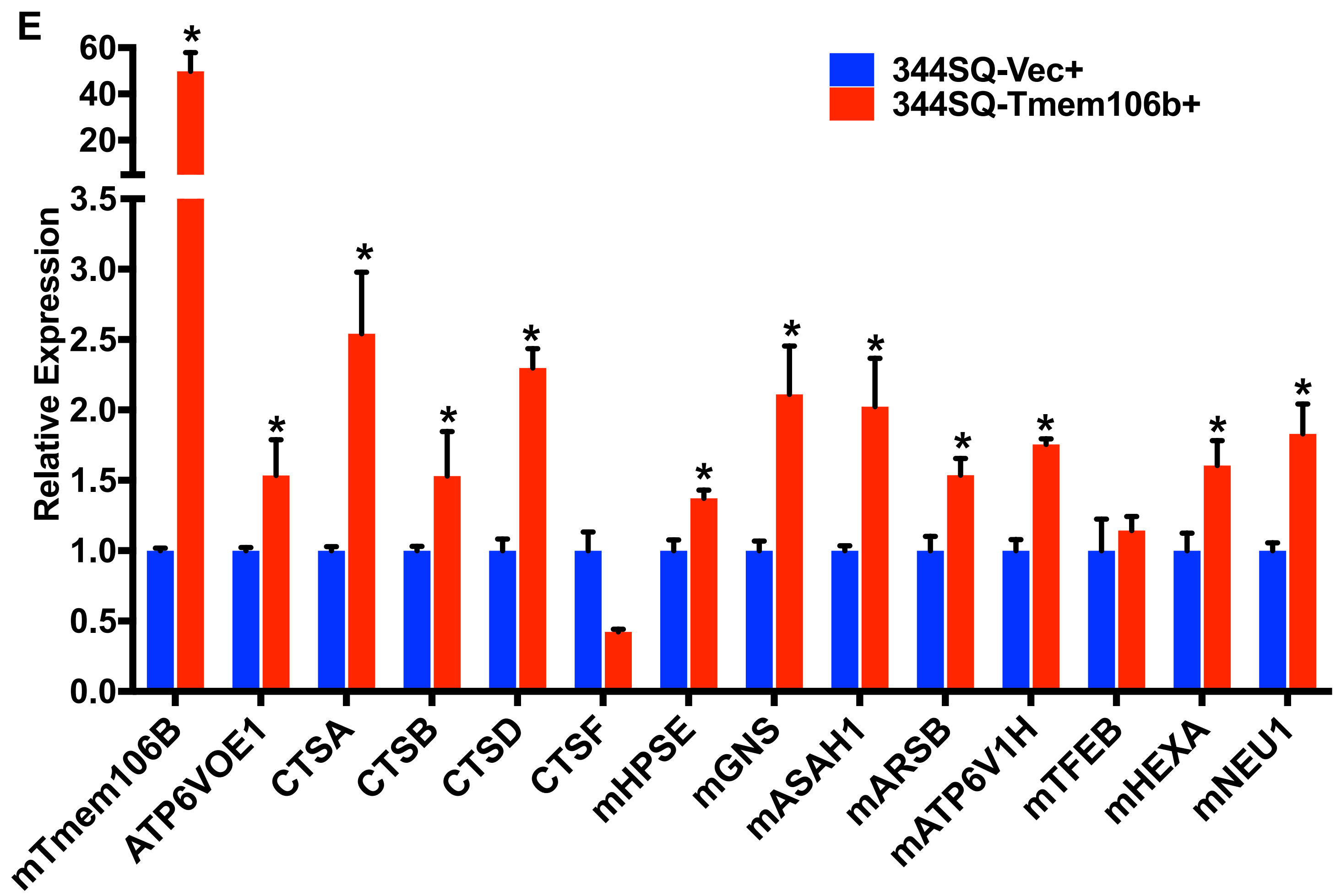

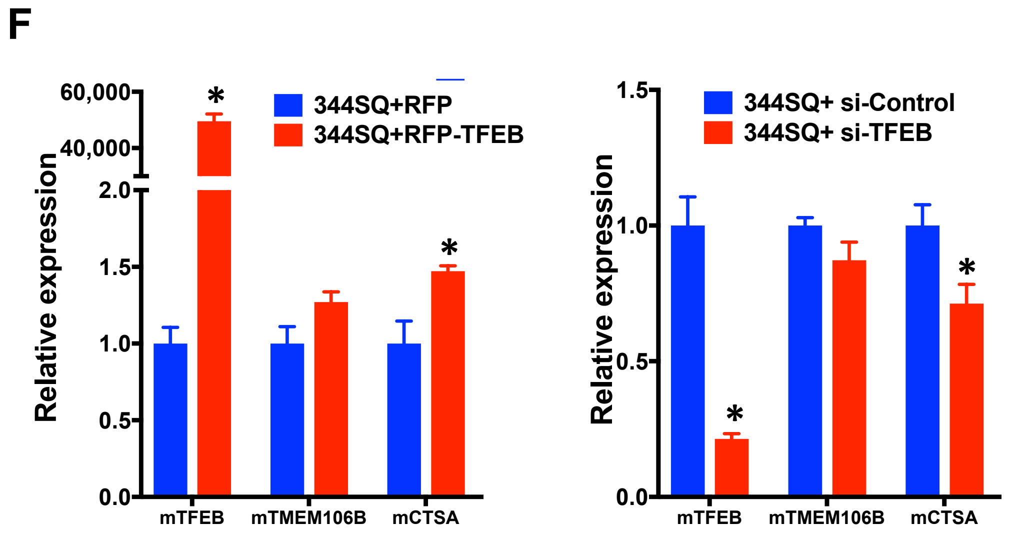

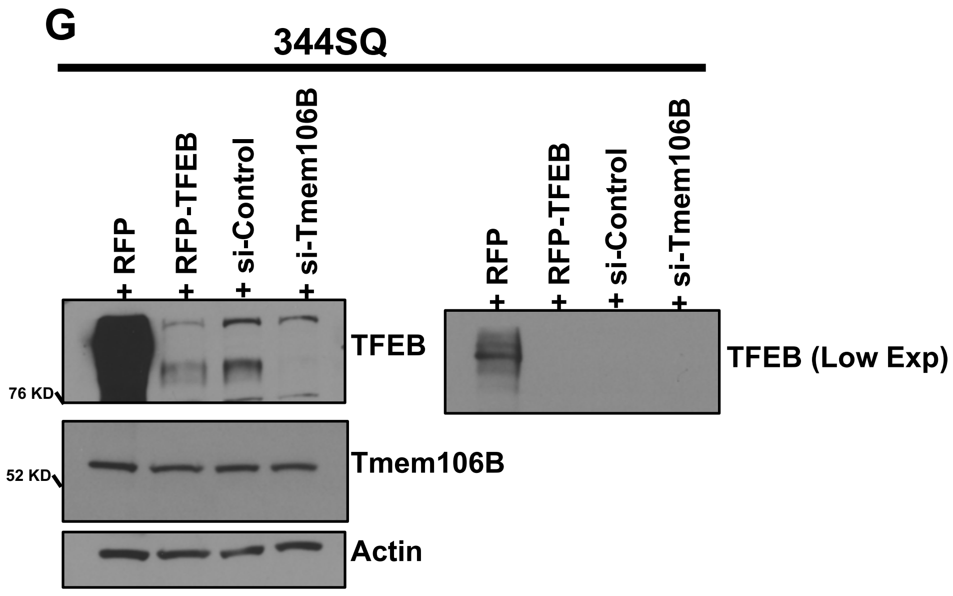

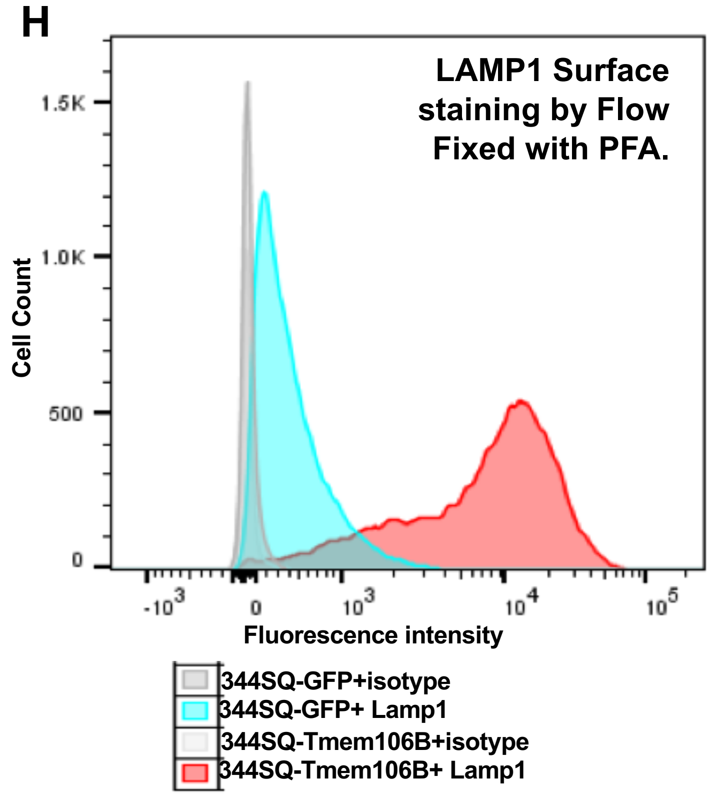

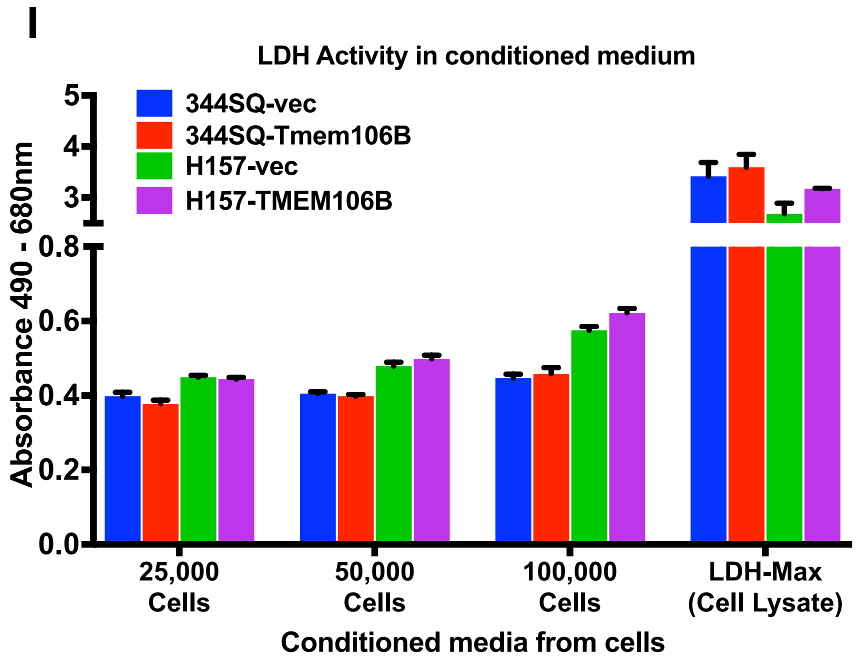

Supplementary Figure 4.

TMEM106B is sufficient but not necessary for the nuclear translocation of TFEB.

(A) Western blot analysis upon sub-cellular fractionation of Human H157 cells with induction of GFP-TMEM106B or GFP alone as control. (B) Western blot analysis upon sub cellular fractionation of cells with induction of GFP-TMEM106B or GFP alone as control after transfection of Mcherry-TFEB construct. Nuclear fractions of cells induced for TMEM106B show increased abundance of TFEB compared to GFP controls. (C) Western blot analysis upon sub-cellular fractionation of 393P cells expressing the different cDNA ORFs for selected in vivo hits, or mCherry alone as control. Nuclear fractions of cells expressing TMEM106B show increased abundance of TFEB compared to other cells. (D) Western blot analysis upon sub-cellular fractionation of 344SQ cells with Scramble shRNA as control or stable expression of shRNA targeting TMEM106B after treatment of different concentrations of Torin1 for 1 hr as indicated. (E) qPCR analysis for expression of lysosomal genes upon induced expression of either GFP only as control or TMEM106B, in mouse 344SQ cells. (F) qPCR analysis of indicated genes after transient over expression or knockdown of TFEB in 344SQ cells. (G) Western blot analysis for TFEB and TMEM106B after transient over expression of RFP tagged TFEB or knock down of TFEB by siRNA transfection. Actin blot as loading control. (H) Flow cytometric analysis of Lamp1 staining on cell surface of 344SQ cells with induction of GFP-TMEM106B or GFP alone. Cells were fixed with PFA and surface stained without permeabilization. (I) LDH activity assay for cytotoxicity in conditioned media from cells induced for GFP only or GFP tagged TMEM106B. All asterisks indicate statistical significance All asterisks indicate statistical significance by T test ($n \geq 3$, * = $p \leq 0.05$).

Supplementary Figure 5

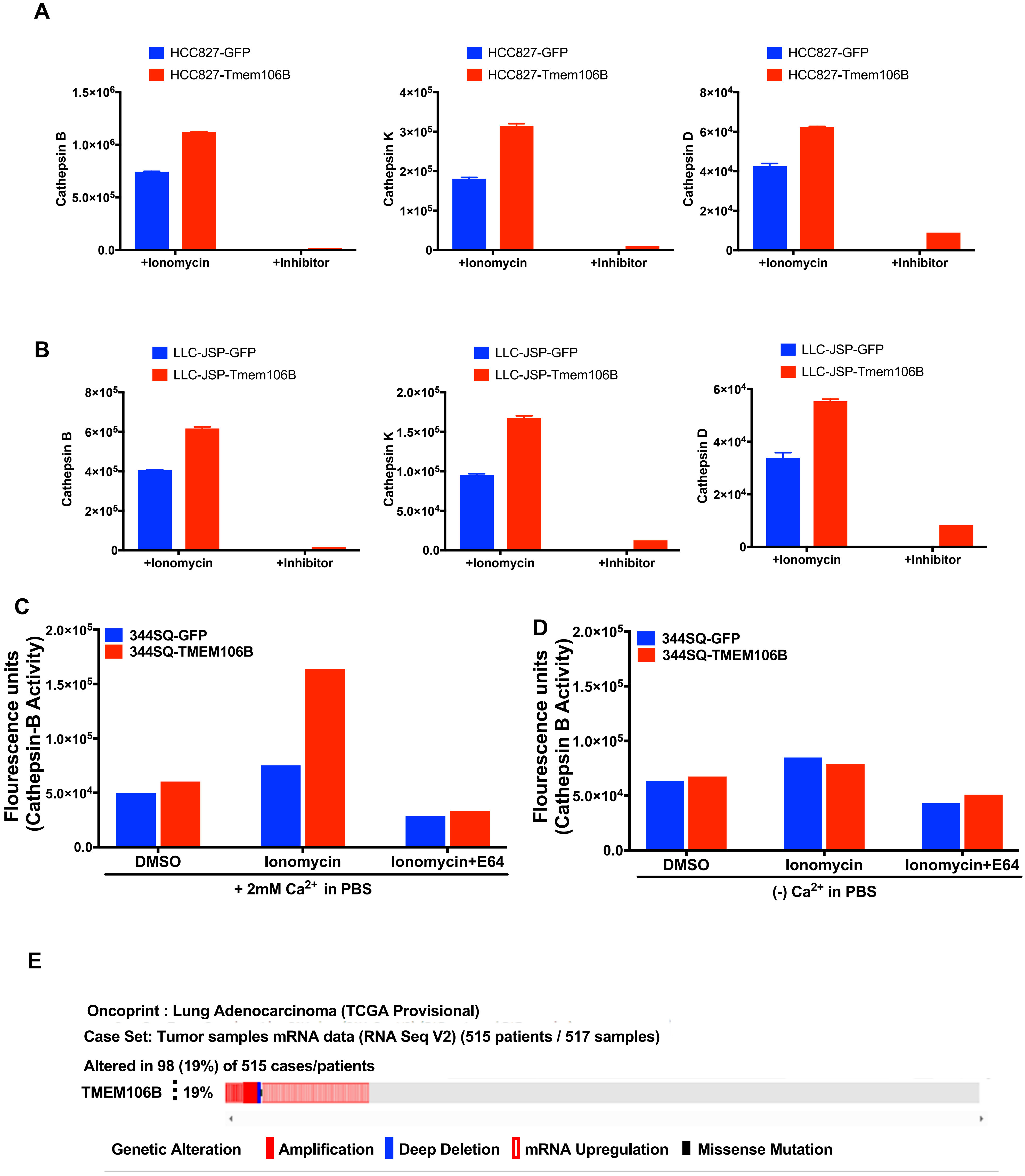

Supplementary Figure 5 contd.

F

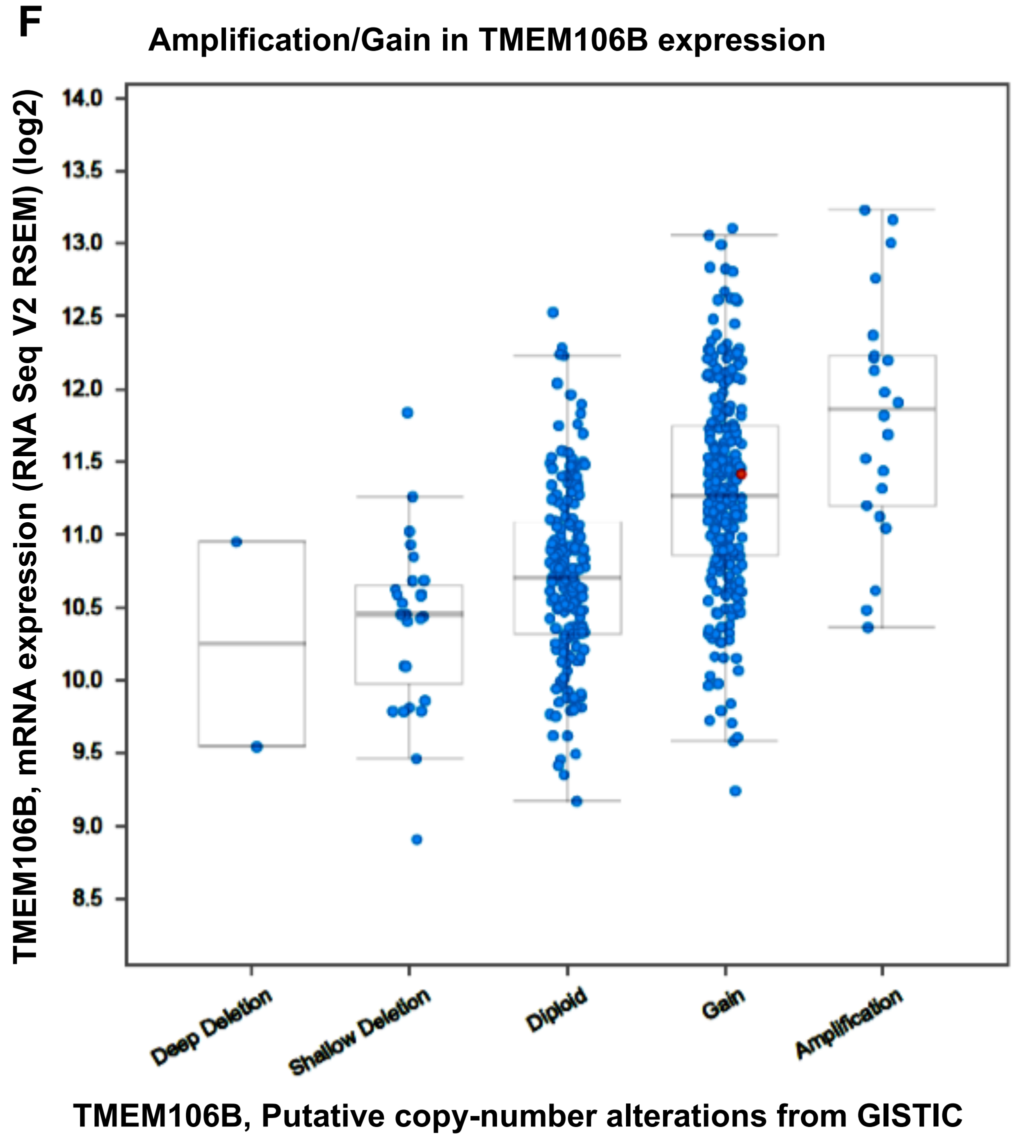

G

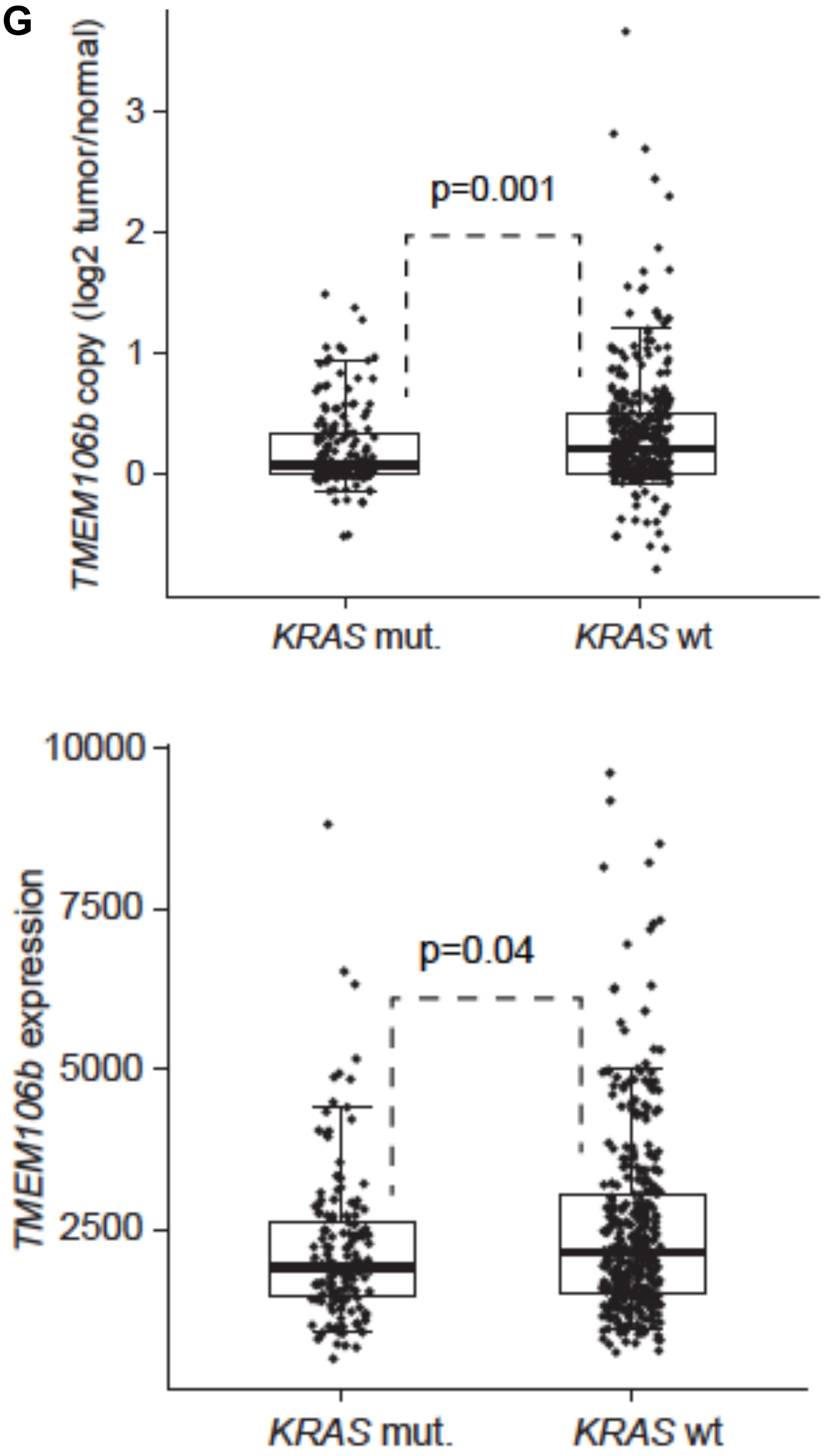

H

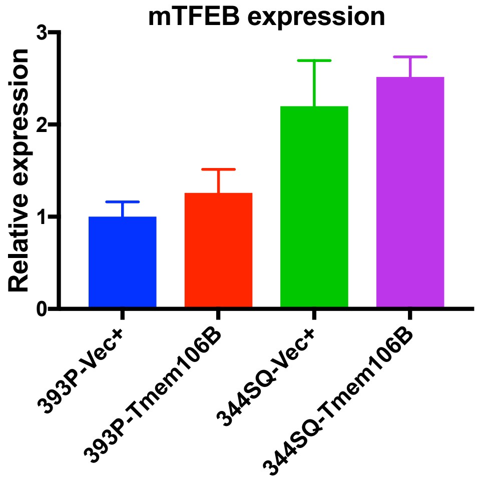

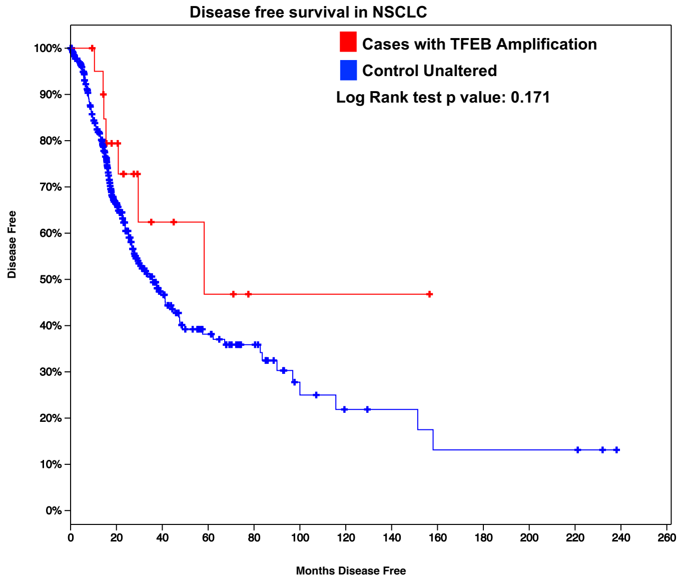

I

Oncoprint : Lung Adenocarcinoma (TCGA Provisional)

Case Set: Tumor samples mRNA data (RNA Seq V2) (515 patients / 517 samples)

Altered in 23 (4%) of 515 cases/patients

TFEB 4%

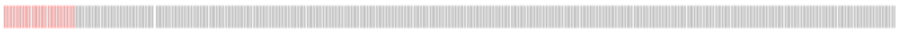

Genetic Alteration mRNA Upregulation No Alteration

Supplementary Figure 5.

TMEM106B induces calcium mediated lysosomal exocytosis in different lung cancer cells and amplification of TMEM106B in human cancers is independent of Kras mutational status.

(A) Activity of cathepsins were assessed using specific activity assays in conditioned phosphate buffered saline (conditioned PBS) where lysosomal contents were released by treating human HCC827 cells or (B) mouse LLC-JSP cells expressing either GFP only or TMEM106B, briefly with ionomycin or DMSO (as control) in the presence of 2mM Ca2+. Assays were also performed in the presence of specific inhibitors, as indicated. (C) Activity of cathepsin-B was assessed using specific activity assay in conditioned phosphate buffered saline (conditioned PBS) where lysosomal contents were released by treating 344SQ cells expressing either GFP only (as control) or TMEM106B, briefly with Ionomycin or DMSO (as control) in presence of Ca2+. Assays were also performed in presence of specific inhibitors as control (as indicated). (D) Activity of cathepsin-B was assessed using specific activity assay in conditioned phosphate buffered saline (conditioned PBS) where lysosomal contents were released by treating 344SQ cells expressing either GFP only (as control) or TMEM106B, briefly with Ionomycin or DMSO (as control) in absence of Ca2+. Assays were also performed in presence of specific inhibitors as control (as indicated). (E) TCGA Onco-Print data for TMEM106B gene expression alterations in Lung Adenocarcinoma (provisional, n=517) data set, showing 19% samples with TMEM106B upregulation. (F) Putative copy-number alterations and gain in TMEM106B gene from GISTIC dataset of TCGA Lung Adenocarcinoma. (G) Top: copy number alteration (log2 tumor : normal) of TMEM106B in human NSCLC samples, according to KRAS mutational status; Bottom: TMEM106B expression, according to KRAS mutational status. KRAS mutation based on "hotspot" mutations 49 as identified in the MC3 exome variant calls from TCGA (https://www.synapse.org/#!Synapse:syn7214402/wiki/405297). (H) qPCR analysis for TFEB expression in cells induced for GFP only or GFP tagged TMEM106B as indicated for 48 hrs. (I) (Left) TCGA Onco-Print data for TFEB gene expression alterations in Lung Adenocarcinoma (provisional, n=517) data set, showing 4% samples with TFEB upregulation. (Right) Disease free survival plots of Lung adenocarcinoma patents with elevated expression of TFEB in TCGA (Provisional, n=517) data set. All asterisks indicate statistical significance All asterisks indicate statistical significance by T test ($n \geq 3$, * = $p \leq 0.05$).

Supplementary Figure 6

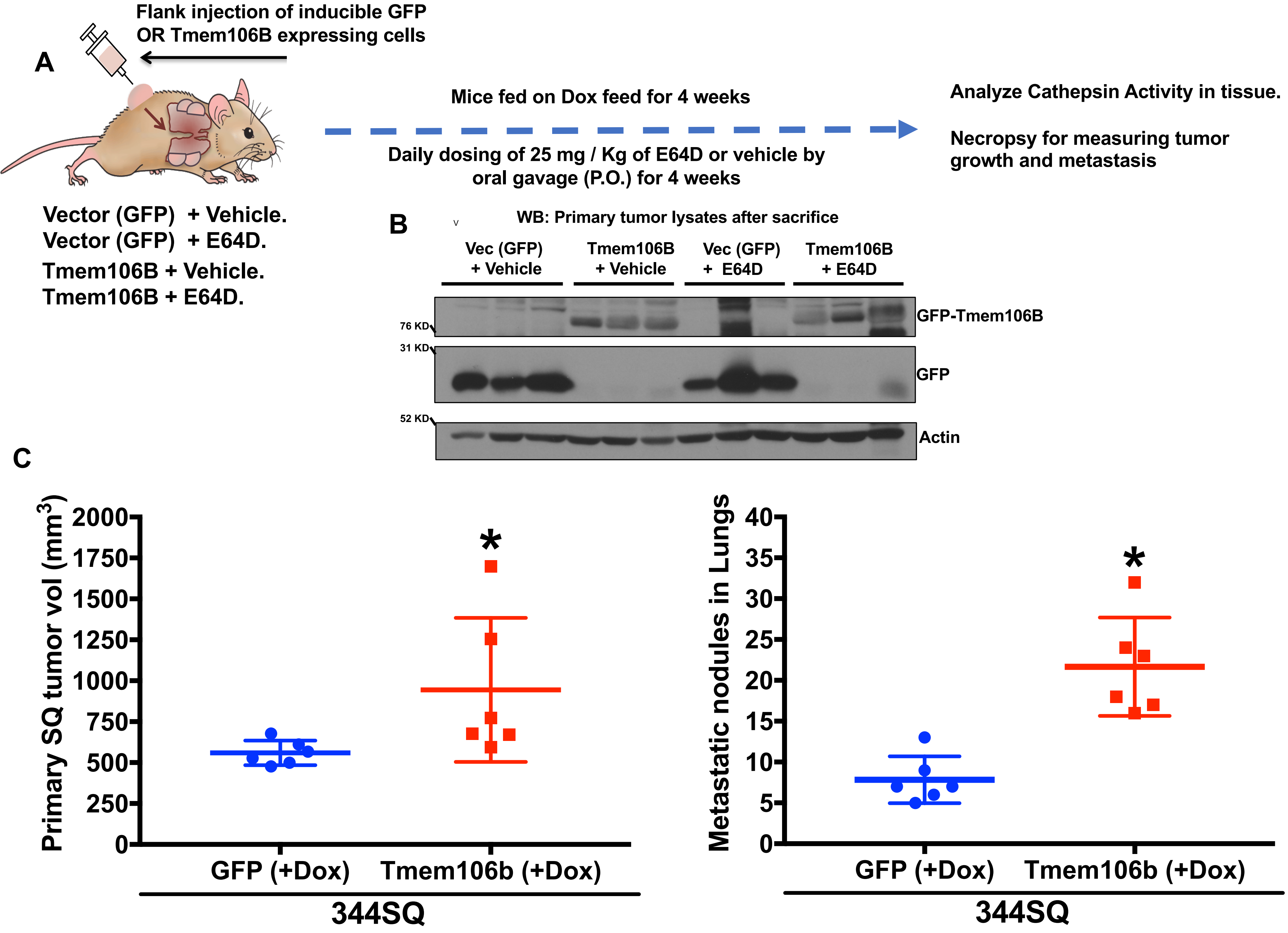

Supplementary Figure 6.

Therapeutic intervention of TMEM106B mediated in vivo metastasis by inhibition of cathepsins.

(A) Schematic representation of experiment plan to test systemic inhibition of Cysteine protease as an effective strategy to prevent TMEM106B mediated metastasis in syngeneic mouse model using TMEM106B inducible cells or GFP only controls. Image created courtesy, elements from open access resource at www.biochem.wisc.edu/medialab/clip-art-media-lab. Contact for more information at medialab@biochem.wisc.edu Phone: 608-262-5116. (B) Western blot analysis to check for induced expression of TMEM106B or GFP only in the representative samples from respective cohorts as indicated. (C) The 344SQ cells with inducible expression of TMEM106B or GFP only as control were injected subcutaneously into syngeneic hosts. Mice were fed with 625 mg/Kg of doxycycline feed for a period of 4 weeks after implantation of the cells. Animals were observed for primary tumor growth and formation of metastatic nodules in lungs after sacrifice. All asterisks indicate statistical significance All asterisks indicate statistical significance by T test ($n \geq 3$, * = $p \leq 0.05$).

Supplementary Table 1

**Primer Name**	**Sequence**
5'Mfe-mTmem106B	GGAGGAACCGGCAATTGATGGGAAAGTCTCTTTCTCACTTACC
3'Xho-mTmem106B	GGCGGGCCTCCTCGAGTTATTGTTGTGGCTGAAGGACATT
5'Hind3-mTFEB	GACTTCAATAAGCTTATGGCGTCACGCATCGGGCTGCGC
3'BamHI-mTFEB	GCTCGGTAAGGATCCTCACAGAACATCACCCTCCTCCATGC
m-ATP6V0E1-F	GGTCCTAACCGGGGAGTTATCATCACC
m-ATP6V0E1-R	AGGCCAGTGGTACTTCAGATACCAGATG
m-CTSA-F	GGAACAGACTTTGGACTTCACTGCAG
m-CTSA-R	CAAGTGTGTCCTCATATCTATGTCTGCC
m-CTSB-F	CTCATGTAGGCTGCTTACCATACACC
m-CTSB-R	CACGCTGTAGGAAGTGTACCCAAAGTG
m-CTSD-F	CTTCTACCTGAACAGGGACCCAGAAGG
m-CTSD-R	CTCATTGCCCACCTCCAACTGGTCCATG
m-CTSF-F	GACCTCACAGAGGAGGAATTCCACACC
m-CTSF-R	CCAAGAGCTCCTGCTCTGACAGGGAG
m-GNS-F	CCTTCAGGAGGAGGTGGCAAACCCTGC
m-GNS-R	CCCTCGAACCAACAGTGGAACTTTG
m-HPSE-F	GGAACTGGGCAATGAGCCCAACAGTTTC
m-HPSE-R	GCCAGCCTTCAGGAAACTCCTCAGC
m-ASAH1-F	CTGGGTATCCTAGAATGGATGTTCG
m-ASAH1-R	GGATCAAGTTCATAGACATCCAAAG
m-TMEM106B-2F	GGCCCACTTGATATGAAGCAGATTGA
m-TMEM106B-2R	CTGCTCAGAGTGTCCAAAGTATGCTG
h-TMEM106B-2F	CACAGTACCTACCGTTATAGCAGAGG
h-TMEM106B-2R	GATACCTCTCCTGGGATATCTGTTCAG
m-TFEB-F	CGCCTGGAGATGACTAACAAGC
m-TFEB-R	GGCAACTCTTGCTTCACCACCT
m-ARSB-F	CACGGTCTTCATCTTCTCCACAG
m-ARSB-R	CGGCTCTTCACGCCTTTCTGTT
m-ATP6V1H-F	GTTGCTGCTCACGATGTTGGAG
m-ATP6V1H-R	TGTAGCGAACCTGCTGGTCTTC

m-GBA-F	GCCAGTTGTGACTTCTCCATCC
m-GBA-R	CGTGAGGACATCTTCAGGGCTT
m-GLA-F	TTGGGCAAGCAGGGCTACTGTT
m-GLA-R	AGGACAAGGTCCACCAATCTCC
m-HEXA-F	TGGCACTTGGTGGACGACTCTT
m-HEXA-R	GCCTTGCGTATTCAATGACCTCC
m-NAGLU-F	GGCTGACACCTTCAATGAGATGC
m-NAGLU-R	AGCAGCCAAACAGCATCAGGGT
m-NEU1-F	CTGTCTCCTCAGTGATGACCAC
m-NEU1-R	ATGACCGAGCCATCTGGAAGCT
m-PSAP-F	GTCTGATGTCCAGACTGCTGTG
m-PSAP-R	CTGGACACAGACCTCGGAATAC
m-SCPEP1-F	TGTGACTGTCCGAAAGGATGCC
m-SCPEP1-R	CCAAATCCAGTGCTAGAACCACC
m-TMEM55B-F	CGTCTGTCAGTCTCCGATCAAC
m-TMEM55B-R	CGGACATACTTCTTTCCTGGAGG
m-TPP1-F	GCGATACAACCTGACAGCCAAAG
m-TPP1-R	AAACTGCCACCGAATAGGCGCA
h-TFEB-F	CCTGGAGATGACCAACAAGCAG
h-TFEB-R	TAGGCAGCTCCTGCTTCACCAC
h-CTSA-F	GCTTCGTGAAGGAGTTCTCCCA
h-CTSA-R	CTGTGGTCATCAGTATGGCTGC
h-CTSB-F	GCTTCGATGCACGGGAACAATG
h-CTSB-R	CATTGGTGTGGATGCAGATCCG
h-CTSD-F	GCAAACTGCTGGACATCGCTTG
h-CTSD-R	GCCATAGTGGATGTCAAACGAGG
h-HEXA-F	GGAGGTCATTGAATACGCACGG
h-HEXA-R	GGATTCACTGGTCCAAAGGTGC
h-NEU1-F	TCCAAGGCTGAGAACGACTTCG
h-NEU1-R	TCAGCAAAGGCGAGAAGAGTGC
h-ATP6V0E1-F	GGTGACCTGTTCAGTTTGCTGC
h-ATP6V0E1-R	GAGCATGTCTTCTTCCTCAAGGC

**Antibodies**	Vendor	Catalogue	Dilution used
TMEM106B	Abcam	ab140185	1:500
TMEM106B	Bethyl	A303-439A	1:1000
β-Actin	Sigma	A2228	1:10,000
TFEB	Bethyl	A303-673A	1:1000
GFP	Santacruz	SC9996	1:1000
Tubulin	Sigma	T9026	1:1000
Lamin A/C	CST (Cell Signaling Technologies)	4777S	1:1000
LAMP1	Abcam	ab25245	1:1000
Integrin α-6	CST (Cell Signaling Technologies)	3750S	1:2000
Cathepsin A	Abcam	ab184553	1:500
Cathepsin B	CST (Cell Signaling Technologies)	31718S	1:500
HEX-A	Santacruz	sc-134577	1:1000
Cathepsin D	Abcam	ab6313	1:500
Mcherry	Abcam	ab167453	1:1000
